# Pre-inoculation water deficit effects on grapevine physiology, *Xylella fastidiosa* titers, and Pierce’s disease progression

**DOI:** 10.1186/s13104-024-06780-1

**Published:** 2024-04-27

**Authors:** Christopher M. Wallis, Zachary Gorman

**Affiliations:** Crop Diseases, Pests and Genetics Research Unit, United States Department of Agriculture-Agricultural Research Service, San Joaquin Valley Agricultural Sciences Center, Parlier, CA 93648 USA

**Keywords:** Drought, Water deficit, Amino acids, Sugars, Phenolics, Plant host resistance, Grapevine (Vitis spp.), *Xylella fastidiosa*, Pierce’s disease

## Abstract

Drought and Pierce’s disease are common throughout many grapevine-growing regions such as Mexico and the United States. Yet, how ongoing water deficits affect infections of *Xylella fastidiosa*, the causal agent of Pierce’s disease, is poorly understood. Symptoms were observed to be significantly more severe in water-stressed plants one month after *X. fastidiosa* inoculation, and, in one experiment, titers were significantly lower in water-stressed than well-watered grapevines. Host chemistry examinations revealed overall amino acid and phenolic levels did not statistically differ due to water deficits, but sugar levels were significantly greater in water stressed than well-watered plants. Results highlight the need to especially manage Pierce’s disease spread in grapevines experiencing drought.

## Introduction

*Xylella fastidiosa* (*Xf*) is a xylem-limited bacterium and results in damaging leaf scorch diseases including Pierce’s disease (PD) of grapevine [1]. In addition to threats from PD, grapevines are expected to face cultivation changes due to lack of water resources [2]. The physiological mechanisms underlying both PD and water deficit stress include synthesis of phenolic metabolites in plants [3, 4]. These phenolics include the compounds flavonoids and stilbenoids, which are antioxidants, alleviate cellular stresses, and form polyphenols that fortify cell walls [5–7]. In addition to phenolics, some amino acids, including proline, are involved in fortifying cell walls to protect plants against both pathogens and drought [8, 9]. Indeed, amino acids and sugars were found to be correlated with *Xf* tolerance in olives [10]. Contrarily, amino acids and free sugars were correlated with susceptibility to PD in grapevine [11]. To complement previous studies that evaluated the impact of simultaneous drought and *Xf* infection on PD development, this study was designed to specifically detail how pre-emptive water deficits affect *Xf* infection and subsequent PD development. Previous studies showed water stress exacerbates *Xf*-induced symptoms and is a central component in PD progression [10, 12, 13]. However, despite correlations between water stress and PD, these stressors cause distinct symptoms when applied separately [12, 13], and PD progression is, at least during the initial stages, unrelated to vascular occlusions [14]. Ultimately, other factors, such as systemic *Xf*-derived toxins or plant-derived signals, likely also facilitate PD [12, 13, 15]. Thus, there remains a need to further examine the interactions between water stress and infections by *Xf*, and examine physiological changes associated with each. Thus, this study measured phenolics, amino acids, and free sugars to better elucidate the mechanisms underlying effects of pre-emptive water stress on *Xf* infection and PD. These results further the understanding of the association between water availability and PD.

## Main text

### Methods

Two separate replicate experiments, in June 2018 or May 2019, were performed to analyze the effect of pre-emptive drought on *Xf* infection and PD. In both experiments, 48 two-year-old ‘Cabernet Sauvignon’ grapevines on ‘101-14MG’ rootstocks were planted in a 1:1 autoclaved field soil: potting mix media, Sunshine Mix #1 from Sungro (Agawam, MA, USA), in 20 L pots and kept in a greenhouse under controlled conditions [4]. After two weeks, 24 grapevines each were either watered to capacity three times a week or exposed to a water deficit to cause water stress. This were placed into two spatial blocks in a completely randomized block design. For the water stressed plants, water was withheld until soil moisture levels dropped below 5%, as monitored by a Watchdog Soil Moisture Sensor (Spectrum Technologies, Aurora, IL, USA), and then kept there an additional minimum of seven days. Pre-dawn water potentials using a Model 615 Pressure Chamber from PMS Instrument Company (Albany, OR, USA) confirmed water stress with measured values below −700 kPa (compared to −400 kPa for well-watered vines). Once the water-stress on the associated grapevines was obtained, 12 well-watered or water-stressed plants were mock-inoculated or pin-pricked inoculated with the Stag’s Leap isolate of *Xylella fastidiosa* subsp. *fastidiosa* (GenBank Accession# LSMJ00000000), a commonly utilized strain [16]. After inoculation, all plants were then kept well-watered to avoid the simultaneous combination of drought and PD. One month after *Xf* inoculation, all plants were photographed to analyze PD symptoms, and 10–20 cm segments of the apical end of a branch were harvested to assess *Xf* titers and analyze metabolites [4], just before the inoculation treatments and at the end of the experiment.

PD symptoms were assessed on a 0–5 scale with 0% damage as “0”. 1–10% leaf damage/necrosis rating as “1”, 10–25% damage rating as “2”, 25–50% rating as “3”, 50–100% damage rating as “4”, and complete plant death/collapse as “5” [17]. Minor damage may be recorded as symptoms even in non-infected control plants because symptoms are rankings of necrosis and not necessarily due to PD [4].

For *Xf* titer and chemical analyses, stem segments were debarked to better isolate xylem tissues and were then ground under liquid nitrogen using a mortar and pestle [4]. DNA was extracted from 100 mg using the Plant DNA Kit from Macherey–Nagel (Allentown, PA, USA). *Xf* titers were then assessed using a QX200 droplet digital PCR EvaGreen Supermix from Bio-Rad (Hercules, CA, USA) and a QX200 droplet digital PCR system (Bio-Rad), also using the primers as present in Wallis et al. [18]. None of the control plants tested positive for *Xf*.

Phenolics were extracted from 100 mg tissue in 1 mL of methanol and sugars and amino acids were extracted from 100 mg tissue in phosphate-buffered saline according to the procedures of Wallis et al. [4]. Methanol extracts then had phenolic compounds analyzed on a Shimadzu (Columbia, MD, USA) LC-20AD high performance liquid chromatography (HPLC) system, equipped with a Ascentis RP C18 column (Sigma-Aldrich, St. Louis, MO, USA), connected to Shimadzu PDA-20AD photodiode array detector, with conditions outlined by Wallis et al. [4]. Peak areas were converted to gram amounts by running standard curves of reference standards within the same compound subclass, such as catechin for flava-3-ols, procyanidin B2 for procyanidins, quercetin glucoside for flavonoid glycosides, or resveratrol for stilbenoids [4]. To measure amino acids, 100 µL of the PBS extract was used in the EZ-FAAST Physiological Amino Acid Kit from Phenomenex (Torrance, CA, USA), and then run on a Shimadzu GC-2010 gas chromatograph utilizing the kit-provided column and flame ionization detection utilizing hydrogen as the carrier gas. Kit instructions were followed and utilized both internal and external standards to identify and quantify compounds [19]. Sugars were analyzed by a Shimazu LC10-AD HPLC, equipped with a Supelcogel H column (Sigma-Aldrich), connected to a Shimadzu RID-10 refractive index detector, with standards of fructose and glucose from Sigma-Aldrich used to make standard curves [19].

IBM (Armonk, NY, USA) SPSS ver. 24 was utilized for all statistical tests with α = 0.05. Non-parametric Kruskal–Wallis tests with follow-up Mann–Whitney U pairwise comparisons were used to determine water-deficit and inoculation treatment effects on symptom expression and *Xf* titers. Analyses of variance (ANOVAs) and least significant differences (LSD) tests determined whether the water deficit-inoculation treatment affected compound amounts. Multivariate analyses or variance (MANOVA) was used to analyze effects on all individual amino acids or phenolics as well, with follow-up ANOVAs and LSDs performed when appropriate [10]. Spatial block effects were included in all statistical models initially and removed if non-significant. Spearman’s correlations determined associations between *Xf* titers or symptoms with compound levels.

### Results

**Fig. 1:**
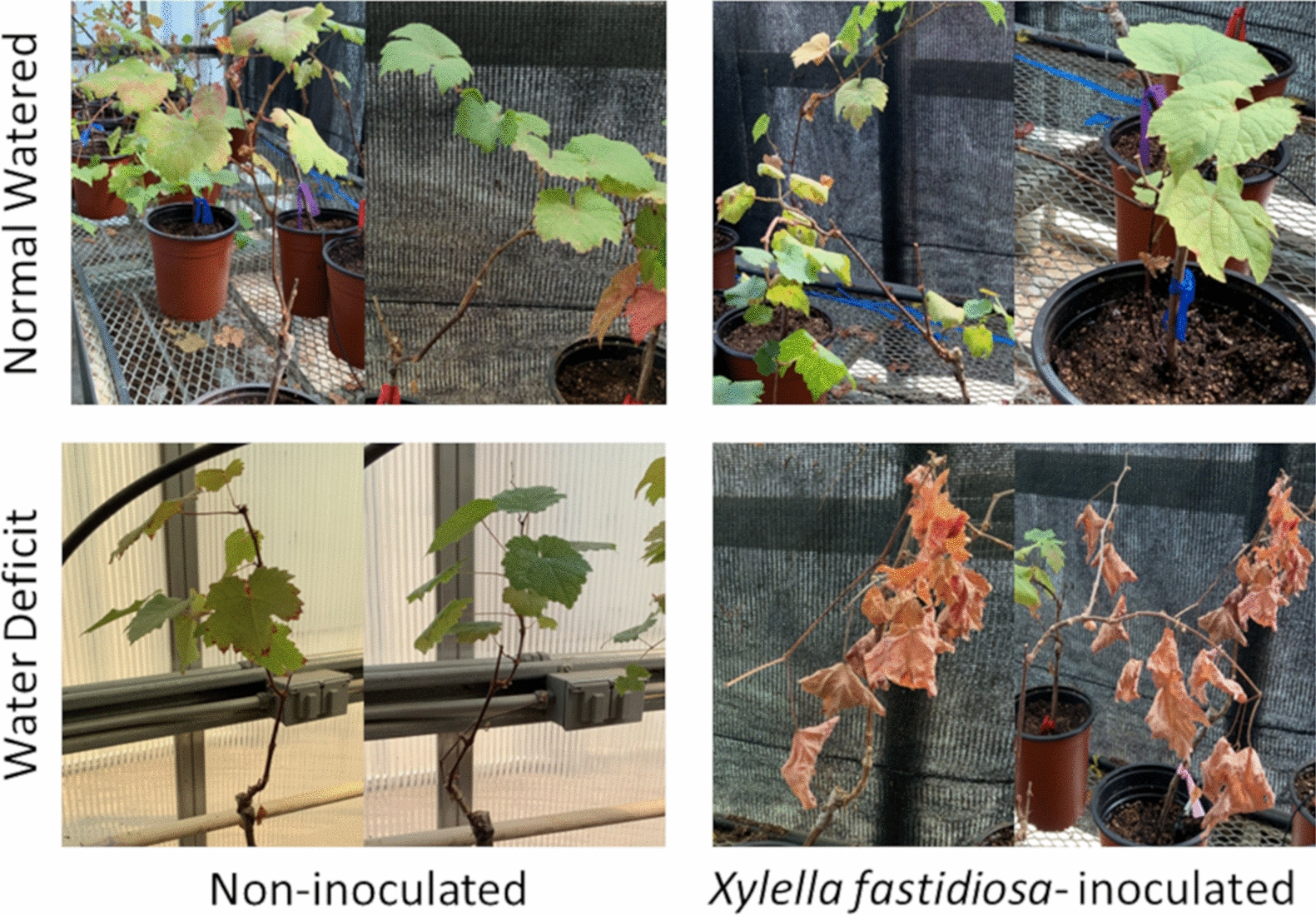
Representative photographs of plants receiving different water or *Xf* inoculation treatments. Two photographs are provided for each treatment combination from the 2019 experiment

**Fig. 2:**
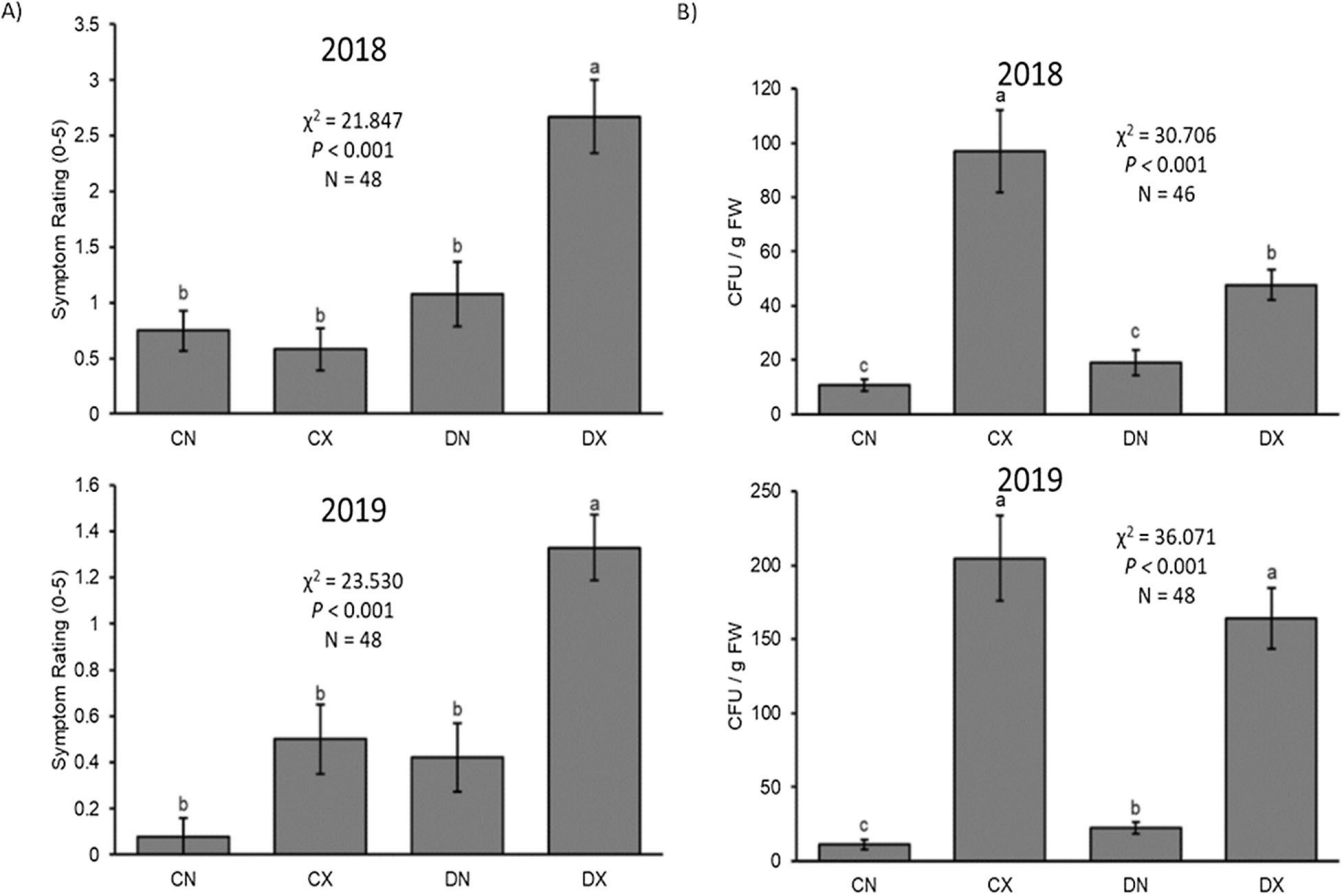
Pierce’s disease symptoms and *Xylella fastidiosa* titers. **A** Mean (± SE) Pierce’s disease symptom ratings (on a 0 to 5 scale) and **B** mean (± SE) *Xylella fastidiosa* titers for the 2018 and 2019 experiments. Kruskal–Wallis statistical test statistics provided. Different letters represent significantly different pairwise comparisons as determined by Mann–Whitney U tests. C = well-watered controls; D = water deficit treated; N = non-infected controls; X = Xf-infected

PD symptoms were significantly greater in *Xf*-infected plants that were pre-emptively drought stressed compared to those that were well-watered (Fig. 1). Well-watered and infected plants displayed mild PD symptoms, which were not statistically different than non-infected plants. Infected well-watered plants had significantly greater *Xf* titers than infected droughted plants in 2018 but not 2019 (Fig. 2). These results suggest that prior water status facilitates PD progression, but inhibits, or at least has no effect on, *Xf* proliferation within grapevine.

**Fig. 3:**
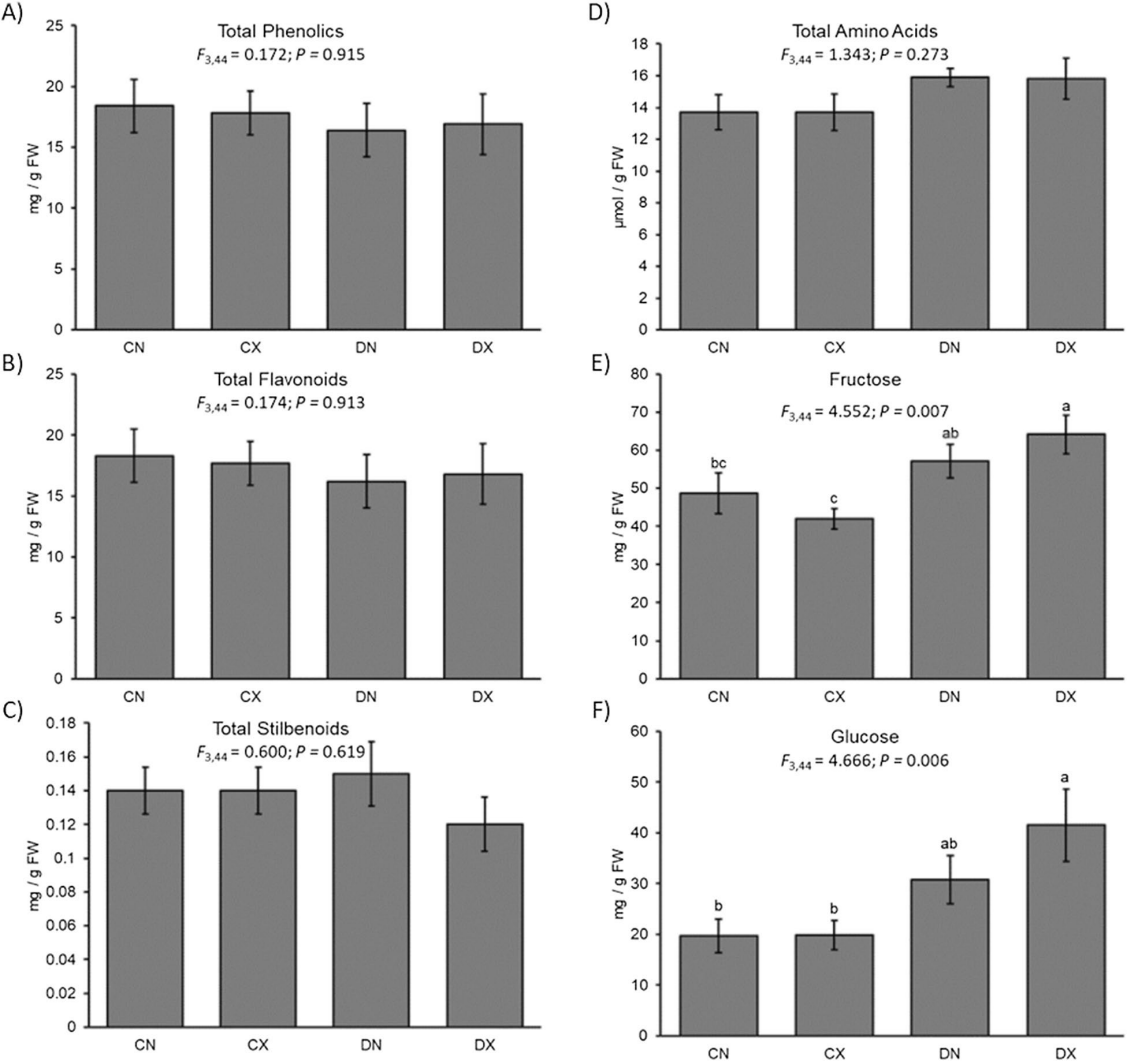
Plant biochemistry levels in response to drought and *Xylella fastidiosa* infection. **A** Mean (± SE) total phenolic levels, **B** mean (± SE) total flavonoid levels, **C** mean (± SE) total stilbenoid levels, **D** mean (± SE) total amino acids levels, **E** mean (± SE) fructose levels, and **F** mean (± SE) glucose levels for the 2019 experiment. ANOVA test statistics provided. Different letters represent significantly different pairwise comparisons as determined by LSD tests. C = well-watered controls; D = water deficit treated; N = non-infected controls; X = Xf-infected

The total amount of phenolics, flavonoids, and stilbenoids did not differ due to water deficit or inoculation status (Fig. 3). MANOVA using the individual phenolic compounds observed a significant effect of treatments (Pillai’s trace = 2.243; *F*_3,44_ = 1.680; *P* = 0.023). Follow-up ANOVAs were significant only for pallidol (*F*_3,44_ = 3.042; *P* = 0.039), and a piceatannol derivative (*F*_3,44_ = 3.088; *P* = 0.037). Levels of pallidol were higher in droughted mock-inoculated plants relative to other treatments, and water deficit treatments induced greater amounts of the piceatannol derivative than well-watered treatments. Pallidol was negatively associated with levels with *Xf* titers (ρ = -0.353; *P* = 0.016; N = 46), and the piceatannol derivative was positively associated with PD symptoms (ρ = 0.404; *P* = 0.004; N = 48).

Total amino acid levels did not differ due to drought or inoculation treatment (Fig. 3). However, MANOVA using individual amino acids observed a significant effect of the treatments (Pillai’s trace = 1.723; *F*_3,44_ = 2.174; *P* = 0.001). Follow-up ANOVAs were significant for alanine (*F*_3,44_ = 3.831; *P* = 0.016; greater in droughted plants compared to well-watered plants), glutamic acid (*F*_3,44_ = 3.623; *P* = 0.020; greater in droughted plants, regardless of infection status, compared to uninfected well-watered controls), phenylalanine (*F*_3,44_ = 2.838; *P* = 0.049; lower in infected plants than in uninfected well-watered controls), tryptophan (*F*_3,44_ = 3.682; *P* = 0.019; lower in all treatments relative to uninfected well-watered controls), and valine (*F*_3,44_ = 4.787; *P* = 0.006; greater in uninfected droughted plants than well-watered plants, regardless of *Xf* infection). Of these amino acids, only phenylalanine levels were negatively correlated with *Xf* titers (ρ = -0.471; *P* = 0.001; N = 46) and symptoms (ρ = -0.331; *P* = 0.022; N = 48).

Analysis of free sugars revealed that fructose levels were greater in *Xf*-infected droughted plants compared to well-watered plants, and greater in uninfected droughted plants relative to well-watered *Xf*-infected plants (*F*_3,44_ = 4.552; *P* = 0.007) (Fig. 3). Glucose levels were greater in droughted *Xf*-infected plants than in well-watered plants, regardless of infection status (*F*_3,44_ = 4.666; *P* = 0.006) (Fig. 3). Both fructose (ρ = 0.289; *P* = 0.046; N = 48) and glucose levels (ρ = 0.324; *P* = 0.025; N = 48) were positively correlated with symptoms but not *Xf* titers.

### Discussion

Previous studies have investigated the role of drought during *Xf* infection and found that drought exacerbates PD symptom development [12, 13]. However, water availability is often temporally dynamic, resulting in fluctuating periods of drought. This necessitated the need to investigate how preemptive drought and recovery affects subsequent *Xf* infection and PD progression. This study has revealed that prior water deficits also can enhance development of PD symptoms upon subsequent *Xf* infection. Conversely, it appeared, albeit not consistently significant, that plants that were previously droughted had lower *Xf* titers by the end of the experiment than those that were previously well-watered. This lack of correlation between PD symptoms and *Xf* titers was also evident by comparing the overall PD scores and Xf titers of the 2018 and 2019 experiments, which show much lower Xf titers, but greater PD scores, in 2018 relative to 2019. Taken together, it seems that while water stress worsens PD symptom development and plant health, it also affects *Xf* proliferation and survival. Though *Xf* titers are usually correlated with symptom severity, Ingel et al. [14] revealed that tylose formation in a particular vessel element is independent of the presence of *Xf* in that vessel. This suggest that tylose formation and symptom development may be caused by additional factors, such as plant or *Xf*-derived systemic signals and/or the amplitude of systemic host grapevine responses to these signals.

Metabolite analysis revealed few differences among different treatments in this study. Phenolic levels appeared only minorly affected by *Xf* infections. However, amounts of several specific amino acids and sugars were significantly altered in response to different combinations of drought and *Xf* infection. For instance, glucose and fructose accumulated to even greater amounts in droughted *Xf*-infected plants than just the droughted plants alone. De Pascali et al. [20] recently found these sugars were associated with resistant to *Xf* in olive. Furthermore, these are known osmoregulatory compounds [21], so it is logical that these metabolites would be elevated in plants that previously lacked adequate water.

In conclusion, the results from this experiment add to the understanding of how drought may impact *Xf* and PD development, emphasizing the potential for prior drought to facilitate PD. These results have particular significance for newly transplanted grapevines in areas that are prone to water shortages. Efforts to ensure adequate watering and vector management in young vineyards should be a priority to prevent mortality caused by *Xf* infections.

## Limitations

- All assessments in this study were taken only once, whereas an expanded time-course would be appropriate in future studies to observe gradual changes in symptoms, titers, and physiology over time.
- The use of different grapevine cultivars, *Xf* strains, and watering regimes would expand upon the finding of this study.
- Additional measurements would be warranted in similar studies such as assessing shifts in transcripts and proteins in different tissues.
- Consideration of adding a water deficit recovery period prior to *Xf* inoculation also could be important to understanding long-term drought stress effects on PD development.

## Data Availability

All data involved with this work is freely and openly available at the U.S. Department of Agriculture-National Agricultural Libraries’ Ag Data Commons at 10.15482/USDA.ADC/1527768.
